# Supplementary material for: Ultrafast phonon-mediated dephasing of color centers in hexagonal boron nitride probed by electron beams
Source: Nat Commun. 2025 Mar 8;16:2326. doi: 10.1038/s41467-025-57584-1 (PMC11890617; doi:10.1038/s41467-025-57584-1)
Supplement: Supplementary file 1 — Supplementary Information [file 41467_2025_57584_MOESM1_ESM.pdf]

Supplementary Information

Ultrafast phonon-mediated dephasing of color centers in hexagonal boron nitride probed by electron beams

M. Taleb[1,2], P. Bittorf[1], M. Black[1], M. Hentschel[3], W. Sigle[4], B. Haas[5], C. Koch[5], P. A. van Aken[4], H. Giessen[3], N. Talebi[1,2,*]

[1]*Institute of Experimental and Applied Physics, Kiel University, 24098 Kiel, Germany*

[2]*Kiel Nano, Surface and Interface Science KiNSIS, Kiel University, 24118 Kiel, Germany*

[3]*4th Physics Institute and Research Center SCoPE, University of Stuttgart, 70569 Stuttgart, Germany*

[4]*Stuttgart Center for Electron Microscopy, Max Planck Institute for Solid State Research, 70569 Stuttgart, Germany*

[5]*Department of physics, Humboldt University, 12489 Berlin, Germany*

E-mail: talebi@physik.uni-kiel.de

Content:

1. **Theoretical Modelling of the CL Emission from the Defect Centers**
2. **Experimental Setup**
3. **Photoluminescence and Cathodoluminescence Spectra of Defect Centers**
4. **Dephasing Dynamics of the Defects Emitting at the Wavelength of 540 nm**
5. **Phonons And Phonon-Polaritons in HBN Flakes**
6. **Excitation of multiple defects and energy-exchange rate between emitters**

Supplementary Note 1. Theoretical Modelling of the CL Emission from the Defect Centers

We propose here a master equation for the theoretical description of the emission from defect centers, when the defects are excited by both a coherent optical pulse and the electron beams.

We first consider only the excitation of the system by electron beams. The density matrix of an N-level quantum system interacting with electron beams can be modeled as[1,2]

$$\frac{d\hat{\rho}}{dt} = -\frac{i}{\hbar}\left[\hat{H},\hat{\rho}\right] + \hat{D}_{\mathrm{rad}}\hat{\rho} + \hat{D}_{\mathrm{ex}}\hat{\rho}, \tag{1}$$

where $\hat{\rho}$ is the time-dependent density-matrix operator, $\hat{H}$ is the system Hamiltonian described as $\sum_{n=1}^{N}\hbar\omega_n |n\rangle\langle n|$, $\hbar$ is the reduced Planck's constant. The Lindblad radiative decay operator is described as

$$\hat{D}_{\mathrm{rad}}\hat{\rho} = \sum_{m<n}\gamma_{mn}\left(\hat{\sigma}_{mn}^{-}\hat{\rho}\hat{\sigma}_{mn}^{+} - \frac{1}{2}\left\{\hat{\sigma}_{mn}^{+}\hat{\sigma}_{mn}^{-},\hat{\rho}\right\}\right), \tag{2}$$

and the excitation operator for electron beams is given by

$$\hat{D}_{\mathrm{ex}}\hat{\rho} = \sum_{m<n}g_{nm}\left(\hat{\sigma}_{mn}^{+}\hat{\rho}\hat{\sigma}_{mn}^{-} - \frac{1}{2}\left\{\hat{\sigma}_{mn}^{-}\hat{\sigma}_{mn}^{+},\hat{\rho}\right\}\right). \tag{3}$$

Here, γ_{mn} is the radiative decay rate from the state $|n\rangle$ to $|m\rangle$, g_{mn} is the electron-beam excitation rate from the state $|m\rangle$ to $|n\rangle$, and $\hat{\sigma}_{mn}^{+} = |n\rangle\langle m|$ and $\hat{\sigma}_{mn}^{-} = |m\rangle\langle n|$ are the excitation and annihilation operators. Considering the electron-beam current in the nanoampere range as used in our experiments, the value of the excitation rate is on the order of $10^8\ \mathrm{s}^{-1}$ to $10^9\ \mathrm{s}^{-1}$.

The equation of motion (Supplementary Eq. (1)) for a two-level system can be treated semi-analytically, providing some insights into the properties of CL emission from two-level systems. First, Supplementary Eq. (1) is rewritten for the diagonal terms of the density matrix as

$$\begin{aligned}\frac{d\rho_{11}}{dt} &= -g\rho_{11} + \gamma\rho_{22} \\ \frac{d\rho_{22}}{dt} &= +g\rho_{11} - \gamma\rho_{22}\end{aligned}, \tag{4}$$

with the obvious result $\frac{d}{dt}\left(\rho_{11}+\rho_{22}\right)=0$, which denotes the conservation of carriers in the system. Second, the coupled system of equations in (S.4) cannot be treated analytically. Within the linear-response approximation, one can assume that $\rho_{11}=1$, and obtain the first approximation as $\frac{d\rho_{22}}{dt} + \gamma\rho_{22} = +g$, which results in the steady-state response[3]:

$$\rho_{22}(\omega) = \frac{g\tau}{-i\omega\tau+1}, \tag{5}$$

where $\tau = \gamma^{-1}$ is the radiation life time of the two-level system. Since the excitation by the electron beam is initially incoherent, it does not produce a coherent superposition of the ground and excited states. This can be observed by the fact that the time-dependent equation of motion for the off-diagonal terms of the density matrix is given by

$$\frac{d\rho_{12}}{dt} = \left(i\omega_t - \frac{1}{2}(g+\gamma) \right)\rho_{12} = \frac{d\rho_{21}^*}{dt}, \tag{6}$$

which leads to the solution $\rho_{12} = \rho_{12}(0)\exp\left(\left(i\omega_t - \frac{1}{2}(g+\gamma)\right)t\right)$, with $\omega_t = \omega_2 - \omega_1$, and is completely decoupled from the diagonal elements of the density matrix. Therefore, if the initial values of the coherence terms are zero, i.e., $\rho_{12}(0) = \rho_{21}(0) = 0$, the coherence terms will remain zero. However, the situation changes completely when the system is initially prepared in a superposition by the EDPHS radiation, contributing to the coherent part of the CL emission, as will be discussed later.

For a pure electron beam excitation, the time-dependent CL emission is theoretically modeled by the expectation value of the photon number, given by

$$\langle \hat{n}(t) \rangle = \mathrm{tr}\left\{ \hat{\sigma}^+ \hat{\sigma}^- \hat{\rho}(t) \right\}, \tag{7}$$

where $\hat{n} = \hat{\sigma}^+\hat{\sigma}^-$ is the photon number operator. The equations of motion for the time-dependent annihilation and creation operators are given by $\frac{d\hat{\sigma}^{\pm}}{dt} = -\frac{i}{\hbar}\left[\hat{H}, \hat{\sigma}^{\pm}\right]$, giving $\sigma^+ = \sigma^+(0)e^{-i\omega_t t}$ and $\sigma^- = \sigma^-(0)e^{+i\omega_t t}$. Substituting this into equation (S.7) and taking the Fourier transform, we obtain the spectral density function[4]

$$S(\omega) = \mathrm{Re}\int_0^{+\infty} d\tau\, e^{-i\omega\tau} \int_{-\infty}^{+\infty} dt \sum_{m<n} \mathrm{tr}\left\{ \hat{\sigma}_{mn}^+(t)\hat{\sigma}_{mn}^-(\tau - t)\rho(t) \right\}, \tag{8}$$

For a two-level system, Supplementary Eq. (8) is rewritten as

$$\begin{aligned} S(\omega) &= \mathrm{Re}\int_0^{+\infty} d\tau\, e^{i(\omega-\omega_t)t}\rho_{22}(t) = \mathrm{Re}\left\{ \tilde{\rho}_{22}(\omega - \omega_t) \right\} \\ &= \mathrm{Re}\frac{g\tau}{-i(\omega-\omega_t)\tau + 1}, \end{aligned} \tag{9}$$

where Supplementary Eq. (5) is used. The right-hand side of Supplementary Eq. (9) has a Lorentzian form, representing the Lorentzian spectral shape of the transition and the recorded CL spectra. Note that in contrast to coherent laser excitation, where the induced polarization in the two-level system results in the generation of coherent light, electron-beam excitation produces light in the number state, as can be seen from Supplementary Eq. (6), which gives a zero value for the coherence terms of the density matrix. In the case of electron beam excitation of a two-level system, the expectation value of the field itself vanishes, while the intensity of the generated light exhibits a Lorentzian line shape.

The generalization of the above solutions to the N - level system is straightforward and is treated numerically using the Runge-Kutta method. We note that by choosing $N = 8$, a good agreement with the experimental results is observed, for both the Experiments 1 and 2 scenarios described in Figure 1 of the main text. In particular, a good agreement between the measured CL spectrum and the $S(\omega)$

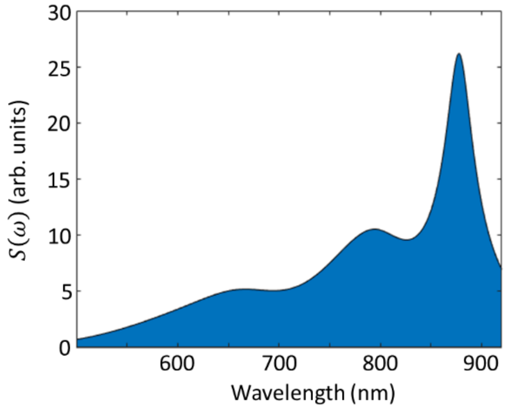

Supplementary Figure 1: The spectral density function for a 8-level system, with the parameters depicted in the text.

is obtained (Supplementary Fig. 1), when $g_{12} = 9\times10^{8}\,\mathrm{s}^{-1}$, $g_{13} = 8\times10^{8}\,\mathrm{s}^{-1}$, and $g_{nm} = 5\times10^{8}\,\mathrm{s}^{-1}$ for all remaining transitions. For the decay rates, we consider $\gamma_{12} = 3\times10^{13}\,\mathrm{s}^{-1}$, $\gamma_{13} = 6\times10^{13}\,\mathrm{s}^{-1}$, and $\gamma_{mn} = 4\times10^{13}\,\mathrm{s}^{-1}$ for all remaining states. Furthermore, the transition wavelengths from the first state to all higher energy states are given by 878 nm, 797 nm, 770 nm, 670 nm, 650 nm, 630 nm, 610 nm, 590 nm, in descending order. Obviously, the overall decay rate for an observed peak λ_m is a race between the quantum-path interferences and the transitions from all states to the *m*th state under consideration and, consequently, the transition from the *m*th state to the ground state. For example, while the radiation decay from the second excited state to the ground state is $\tau_{12} = \gamma_{12}^{-1} = 33\,\mathrm{fs}$, the broadening of the peak at 878 is about 3 times narrower, as expected for such an ultrafast decay rate.

Now we consider the case where the quantum system is also excited by the EDPHS radiation. In this case, the Hamiltonian of the system is changed to $\sum_{n=1}^{N}\hbar\omega_n |n\rangle\langle n| - \hat{\mu}\cdot\vec{E}(t)$, where $\hat{\mu}$ is the dipole transition matrix of the 8-level quantum system. To model the response of the system to both excitations, we consider an ultra-broadband EDPHS radiation with the peak electric field amplitude of $10^{8}\ \mathrm{V\,m}^{-1}$ and the broadening of 5 fs. The elements of the dipole transition matrix are considered as $\mu_{12} = 35\,\mathrm{D}$, $\mu_{13} = 85\,\mathrm{D}$, $\mu_{14} = 95\,\mathrm{D}$, $\mu_{15} = 105\,\mathrm{D}$, $\mu_{16} = 115\,\mathrm{D}$, $\mu_{17} = 120\,\mathrm{D}$, $\mu_{18} = 125\,\mathrm{D}$, and $\mu_{mn} = 15\,\mathrm{D}$ for all other transitions, given in Debye unit. In this case, there is a second contribution to the observed CL spectrum, in addition to the spectral density function $S(\omega,\tau)$, which is given by the coherent induced polarization in the system given by

$$P(\omega,\tau_{01},\tau_{02}) = \frac{ne^{2}}{3\varepsilon_0\hbar}\int_{-\infty}^{+\infty} dt e^{-i\omega t} tr\left\{\hat{\mu}\hat{\rho}(t,\tau_{01},\tau_{02})\right\} \qquad (10)$$

where n is the number of emitters (here we consider $n = 1$), τ_{01} and τ_{02} are the delays corresponding to the arrival time of the EDPHS and electron-beam excitations, with the respect to the elapsed time t. The delay in the experimental setup corresponds to $\tau = \tau_{02} - \tau_{01}$. Supplementary Fig. 2 depicts the calculated polarization (Supplementary Eq. (9)), with $\tau_{01} = 50\,\mathrm{fs}$ and varying τ_{02} from 0 to 100 fs. Obviously, before the EDPHS excitation, the polarization response does not show any spectral

interference fringes, whereas after the interaction of the system with the EDPHS, the system is prepared in a coherent superposition

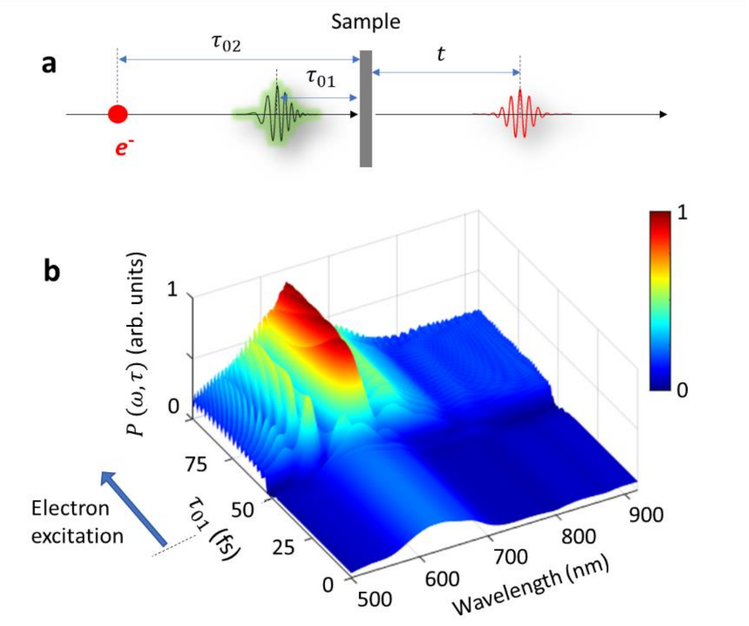

Supplementary Figure 2: Coherent polarization after interaction with the EDPHS radiation. (a) A sample consisting of a 8-level quantum system interacting with the EDPHS radiation arriving at the delay time τ_{01} and with a moving electron arriving at the delay time τ_{02}, with respect to the elapsed time t. (b) Induced polarization in a 8-level quantum system interacting with both the EDPHS radiation and the electron beam excitation, versus the wavelength and τ_{01}, when $\tau_{02} = 50\,\mathrm{fs}$.

resulting in spectral interference fringes. The visibility of the interference fringes in this case is dominant only within the decoherence time scale determined by the dephasing time $T_2 = 2(g+\gamma)^{-1} \simeq 2\gamma^{-1}$ (see Supplementary Eq. (6) and the discussion below), as shown in Figure 3 of the main text.

The states associated with phonon resonances are not energetically equidistant, which is expected due to the anharmonic potential constraining molecular vibrations (described by the polynomial expansion or Morse potential[5,6]). The eigenenergies of an anharmonic oscillator are generally expressed as $E(n) = -E_0 + \hbar\omega_0\left(n+\frac{1}{2}\right)\left\{1-\xi\left(n+\frac{1}{2}\right)\right\}$, where ξ and E_0 are parameters controlling the anharmonicity of the potential and ω_0 is the oscillation frequency of the oscillator. We first find that by choosing $E_0 = 0.090\,\mathrm{eV}$, $\hbar\omega_0 = 0.119\,\mathrm{eV}$, and $\xi = 0.012$, the obtained energies closely match our theoretically predicted eigenstates for an 8-level system (Supplementary Fig. 3a). Moreover, the minimum potential energy of the harmonic oscillator associated with the excited electronic state with respect to the ground electronic state is calculated to be 1.3528 eV. These eigenstates are used in the theoretical model to reproduce three different sets of experimental data: CL spectra, incoherent CL spectra confirming the population decay, and coherent CL spectra obtained by angle-resolved CL spectroscopy.

To justify the choice of 8 states, we present the spectra of incoherent CL excitation (Supplementary Eq. (8)) for different cases where $N = 3$ states and $N = 6$ states are considered (Supplementary Fig. 3). It is evident that the inhomogeneous broadening associated with higher energy states (see Supplementary Fig. 1 and main text) is only accurately reproduced when a minimum of 6 states is considered. Moreover, the induced polarization captures the interference phenomena observed in the CL momentum-resolved spectra as a function of the delay between EDPHS radiation and electron-beam excitation (Supplementary Fig. 3d) when 6 states are considered. However, both the spectral shape and the dephasing time are more accurately reproduced when 8 states are considered. For a system with only 6 quantum states, the dephasing time is significantly longer (Supplementary Fig. 3d).

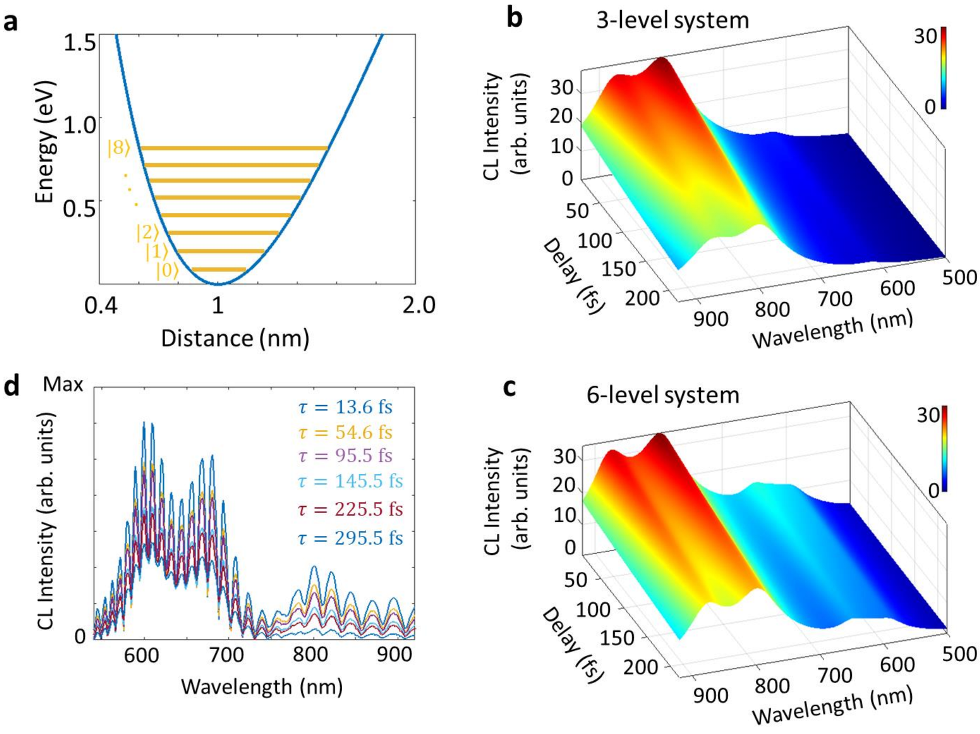

Supplementary Figure 3: Quantum states and calculated CL spectra associated with an *N*-level quantum system. (a) The potential and eigenenergies of an anharmonic oscillator. See text for details. Incoherent CL intensity versus wavelength and delay between the EDPHS and electron excitation for (b) maximum number of 3 states, and (c) maximum number of 6 states. (d) CL intensity associated with the induced polarization versus wavelength at the shown delays between the EDPHS radiation and the electron-beam excitation.

Supplementary Notes 2: Experimental Setup

Our sequential CL spectroscopy setup uses a piezo stage nano-positioner that holds the EDPHS structure on top of the sample holder at a precise distance with respect to the sample stage (Supplementary Fig. 4a). The electron first interacts with the EDPHS structure, resulting in the generation of plasmon polaritons in the EDPHS gold film[7-9]. The EDPHS film consists of a thin gold film of 40 nm thickness on a Si_3N_4 membrane of 30 nm thickness. The plasmon polariton wave propagates at the surfaces of the gold film, where the quasi-symmetric mode couples better to the incident electron beam and also sustains a longer propagation length. The quasi-symmetric polaritonic wave gradually contributes to the radiation continuum by interacting with the implemented nanopinholes. The positioning of the nanopinholes and their radii are designed in a way to enable the generation of a collimated light beam (Supplementary Fig. 4b). In addition, the CL radiation from the EDPHS is ultrabroadband, covering the wavelength range from 560 nm to 940 nm (Supplementary Fig. 4c), corresponding to a temporal broadening of 0.9 fs.

The measurements are performed by acquiring the CL spectra, angular maps, and momentum-resolved spectroscopy at various distances between the sample and EDPHS, where the latter corresponds to the delays between the electron and EDPHS radiation arriving at the sample. The zero point of the delay axis is calibrated by determining the time at which significant changes in the sample response are observed. The EDPHS structure used here is significantly smaller than the EDPHS structure previously used by us to resolve the coherence time of exciton polaritons[10]. Therefore, the EDPHS structure here has a shorter pulse duration and the emission onset from the EDPHS occurs on shorter time scales.

At each distance between the EDPHS and the sample, the electron beamis focused on the hBN flake. Due to the structure of the EDPHS and the position of the nano-positioner on top of the sample, the working distance used in our measurements is long enough to allow a small angular convergence of the electron beam interacting with the EDPHS and the sample. Therefore, for all the EDPHS-to-sample

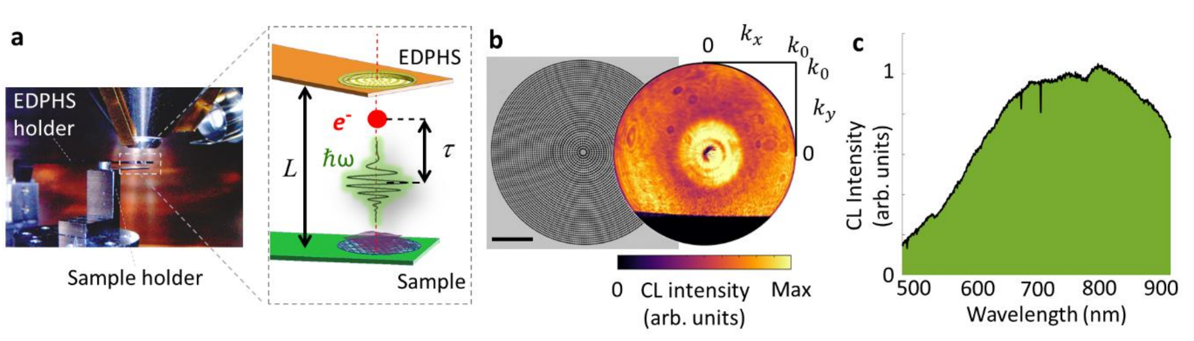

Supplementary Figure 4: Image of the setup for photon-electron correlative spectroscopy. (a) An electron (e^-) with the kinetic energy of $30\ \mathrm{keV}$ is passing an electron-driven photon source (EDPHS) which generates phase-locked photons with a collimated Gaussian spatial profile. Afterwards, electron and photons propagate with different group velocities towards the underlying sample, where the delay τ between their individual arrival times is controlled by the distance L between the EDPHS and the sample via a nano-positioner. The total scattered radiation from the sample is detected and its energy-momentum distribution is analyzed to investigate the phonon-mediated dephasing of the defect centers in hBN. (b) SEM image of the total EDPHS structure with the scalebar equal to $10\ \mu\mathrm{m}$, and an angle-resolved CL measurement of the collimated light generated by the EDPHS versus $k_x = k_0 \sin\theta \cos\varphi$ and $k_y = k_0 \sin\theta \sin\varphi$, where θ and φ are the polar and azimuthal emission angle with respect to the sample plane. The scale bar is $10\ \mu\mathrm{m}$. (c) Integrated CL intensity of the EDPHS structure, demonstrating the ultra-broadband emission from the EDPHS, corresponding to the fractional bandwidth of 50% and central wavelength of 800 nm.

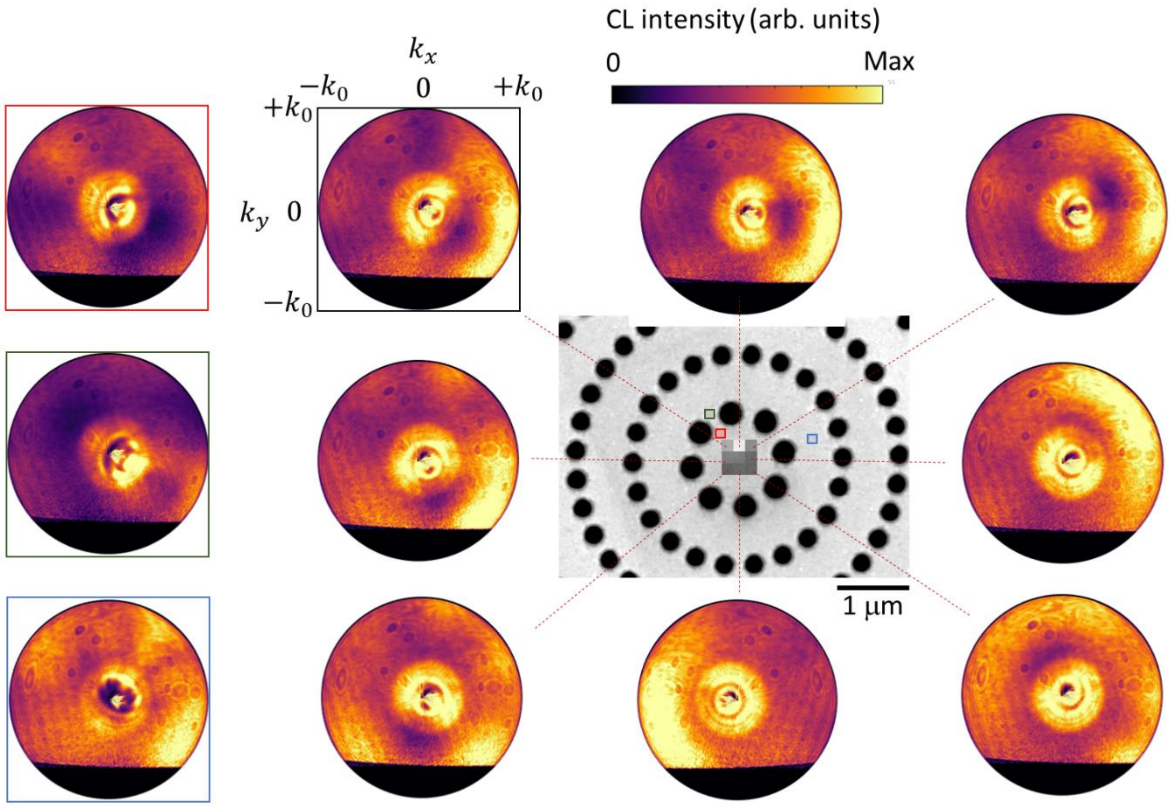

Supplementary Figure 5: Radiation characterization of the electron-driven photon source (EDPHS). Angle-resolved CL measurements of the light produced by the EDPHS upon electron irradiation versus $k_x = k_0 \sin\theta \cos\varphi$ and $k_y = k_0 \sin\theta \sin\varphi$, where θ and φ are the polar and azimuthal emission angles, respectively. Each CL intensity map represents a different position of the electron beam impingement, resulting in additional side lobes in the radiation pattern. The directional pattern is only slightly altered as long as the electron interacts with the sample within the central region. The SEM image shows the structure of the EDPHS and the different electron beam impact positions, connected by dashed lines or color-coded in red, black, and blue for the outer ring.

distances used here, the electron interacts with the EDPHS structure at positions within the inner central ring of the EDPHS (see Supplementary Fig. 5). Within this set of impact positions, the emission from the EDPHS still shows a collimated profile, allowing us to retrieve the momentum coherence of the emitted CL superposition from the sample and EDPHS.

To better demonstrate the impact of EDPHS radiation on the emission from defects, we compare the angle-resolved CL patterns of the defect when irradiated only by electron beams (Supplementary Fig. 6a) to the case when the defect is excited with both electron beams and EDPHS radiation (Supplementary Fig. 6b). The emission is filtered at a wavelength of 850 ± 25 nm. The distance between the EDPHS and the sample is set to 10 µm.

For the former excitation scheme, where only the electron beam interacts with the defect, the emission does not exhibit coherent radiation properties and lacks a dipolar-like emission pattern as expected. This is due to the incoherent interaction of the electron beam with the defect, involving sequential interactions of secondary or backscattered electrons and random emission in various directions.

Upon illuminating the defect with coherent EDPHS radiation, the angle-resolved CL pattern from the defect shows clear interference fringes (Supplementary Fig. 6b), indicative of coherent radiation properties. EDPHS radiation brings the system into a coherent superposition of the excited states, akin to a π/2 pulse in optics. This coherent superposition induces linear polarization within the sample, leading to a coherent CL signal. The coherent component of the CL radiation can then interfere with the EDPHS light, resulting in the observed interference fringes.

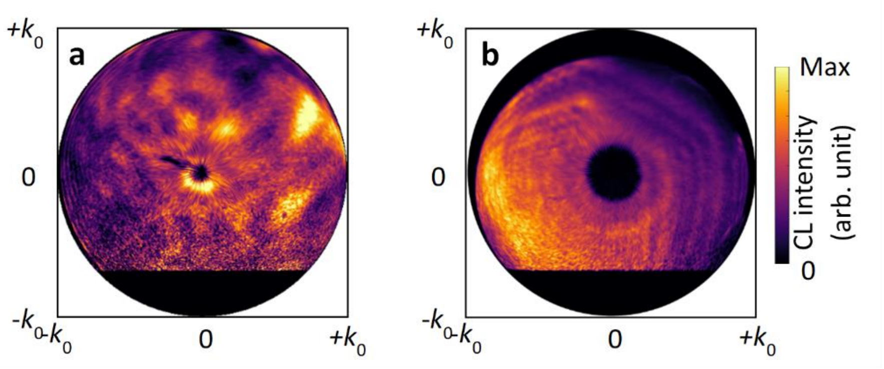

Supplementary Fig. 6: Angle-resolved CL pattern of a defect filtered at the wavelength of 850 ± 25 nm. The defect is excited with (a) electron beams at the kinetic energy of 30 keV, and with (b) both electron beams and EDPHS radiation, when the distance between the EDPHS and sample is set to 10 µm.

Supplementary Note 3. Photoluminescence and Cathodoluminescence Spectra of Defect Centers

Here, a more detailed analysis of the defect centers is performed using CL and PL spectroscopy. Supplementary Fig. 7a shows the SEM image of an hBN flake prepared by liquid exfoliation and positioned on top of a holey carbon TEM grid. The flake has regions of different thicknesses, resulting in the excitation of different emitters, with their emission wavelengths distributed between 570 nm and 880 nm (Supplementary Fig. 7b). Two sharp resonances can be distinguished, which are locally excited by changing the impact position

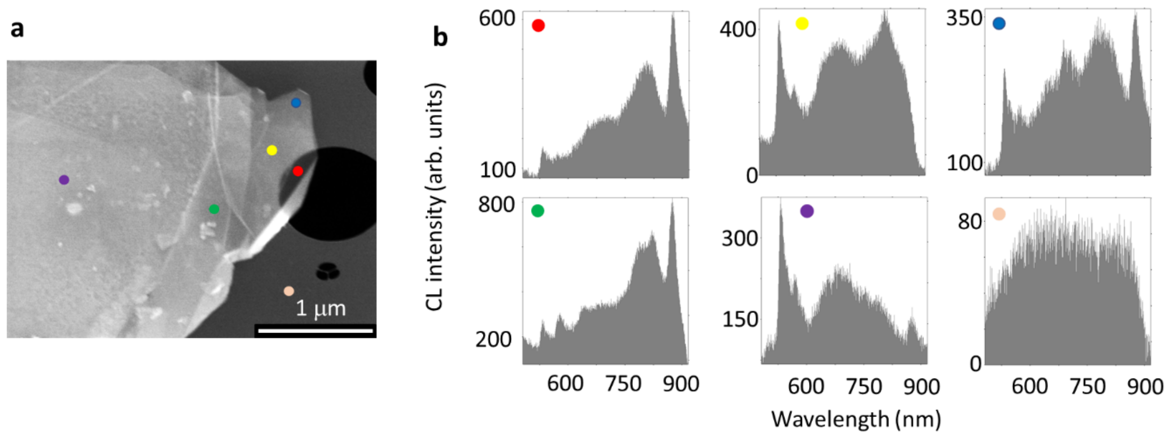

Supplementary Figure 7: Excitation of different atomic defects with electron beams. SEM image of the hBN flake on the holey carbon support film. Each colored point represents a unique impact position of the electron beam. The resulting CL spectra are shown on the right, revealing the individual emission pattern for each excitation spot. The spectra on the bottom right depict the CL response of the underlying carbon film.

of the electron beam. Both emitters couple strongly to the TO phonons and give rise to the phonon sidebands observed in the CL spectrum.

Spectral images acquired at the wavelengths associated with spectral peaks demonstrate the position of defect centers (Supplementary Fig. 8). Particularly, we observe a rather low distribution of the defects emitting at 540 nm. Moreover, these defects have in general a lower brightness compared to the defects emitting at longer wavelengths. This can be attributed to the better coupling efficiency of the electron beams to longer wavelength emitters, due to their electric dipole moment oriented along the direction parallel to the electron beam.

To further clarify the nature of the defects we observe, we additionally perform CL and PL spectroscopy on the same flake ((Supplementary Fig. 9). For the PL measurements, we used a CW green laser with an output power of 180 mW, and used optical density filters to tune the output power. The PL measurements here were performed with an illumination power of 250 μW to avoid damage of the carbon substrate. For our PL measurements, we manipulated the illumination stage of the spectroscopy system provided by New Technologies and Consulting (NT&C).

In the CL measurement, we observe two peaks at wavelengths of 570 nm and 880 nm, where the CL signal associated with the second longer wavelength resonance is prominent. In contrast, the longer wavelength resonance is completely absent in our PL measurements. Together with other investigations based on analytical transmission electron microscopy techniques (see Fig. 2 in the main text), we conclude that the resonance at 880 nm is associated with an electric dipole moment oriented perpendicular to the plane, which couples better to the electron beams.

To better investigate whether trapped charges in defects, caused by the slower secondary electrons emitted from the hBN flake, can lead to the observation of the intense C signal, we additionally perform CL spectroscopy at different acceleration voltages and currents of the electron beams ((Supplementary Fig. 10). In particular, changing the voltage from 5kV to 30 kV decreases the intensity of the CL signal at 880 nm. This is due to the better coupling efficiency of the slower electron beams to the phonon resonances, which is the main mechanism here for the excitation of the emitter. Since no shift in the emission wavelength is observed, we assume that the trapped charges play only a minor role.

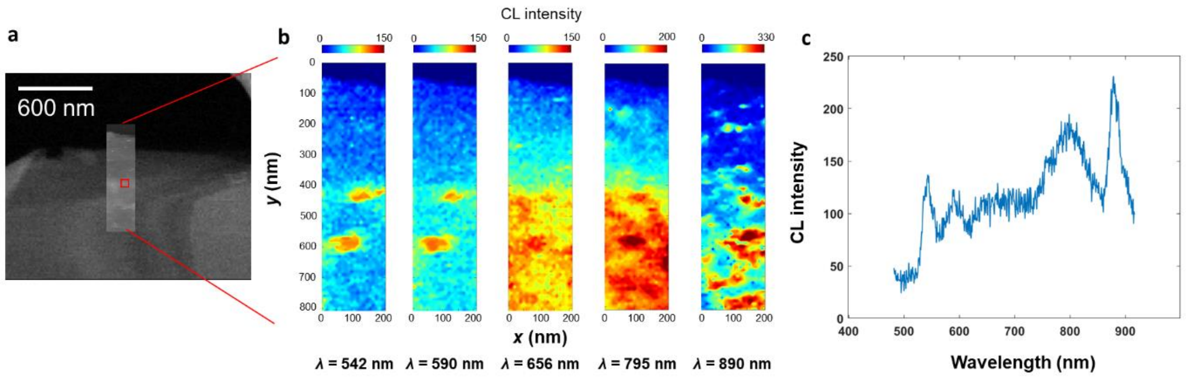

Supplementary Figure 8: Position of different atomic defects. (a) SEM image of the hBN flake on the holey carbon support film. (b) Spectral images shown at depicted wavelengths. (c) CL spectrum at the position marked by the red box on the SEM image.

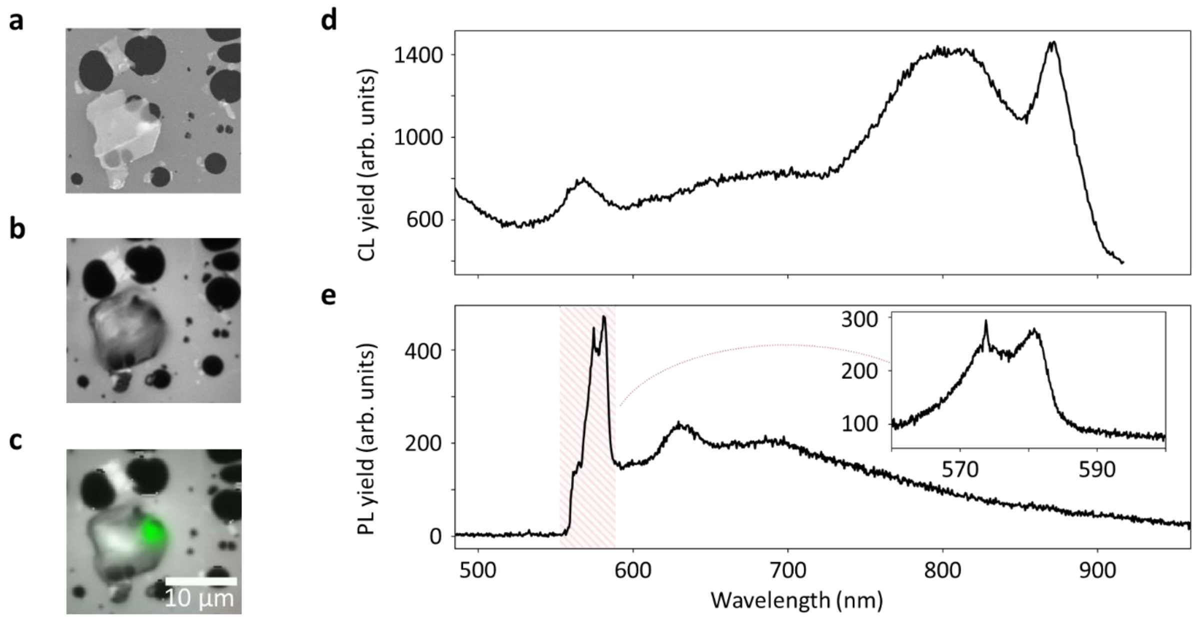

Supplementary Figure 9: Comparison between CL and photoluminescence PL measurements of the hBN flake. (a) SEM image of the hBN flake used in the measurements placed on a holey carbon support film. (b) Optical microscope image of the hBN flake. (c) Optical microscope image of the hBN flake with green laser light illumination. The scale bar is the same for each image. (d) CL spectra of the hBN flake showing emission peaks in the near infrared and around $\lambda = 570\ nm$. (e) PL spectra of the hBN flake featuring emission peaks only in the visible range and missing peaks in the NIR.

Changing the current from 4 nA to 12.7 nA, on the other hand, does not significantly change the emission intensity or wavelength. However, phonon resonances are more pronounced at higher excitation currents.

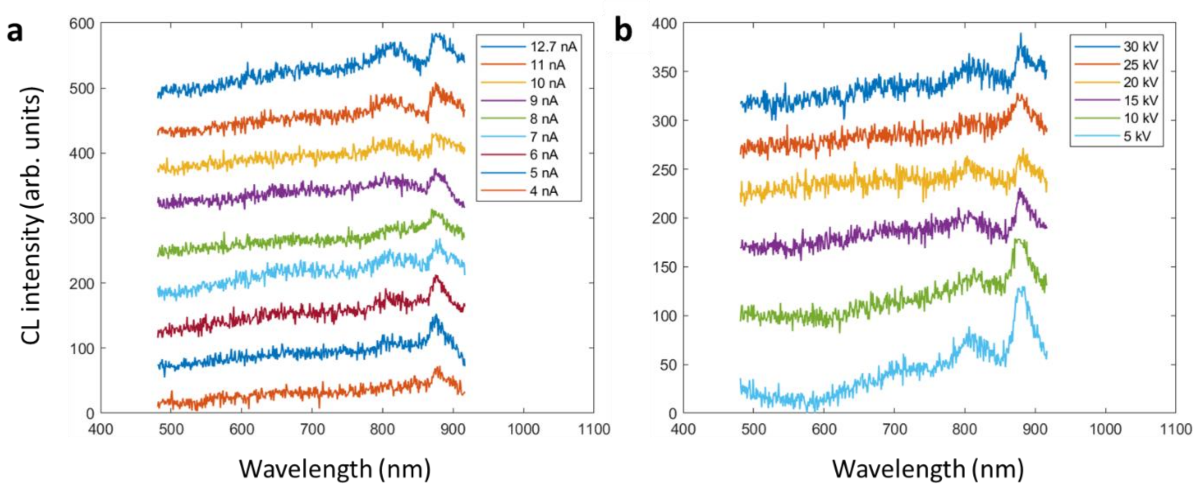

Supplementary Figure 10: Changing electron beam characteristics. CL spectra of the hBN flake displayed in Figure 1 of the main text at different values of (a) beam current and (b) acceleration voltage as indicated in the figure.

Supplementary Note 4. Dephasing Dynamics of the Defects Emitting at the Wavelength of 530 nm

The peculiar ultrafast dephasing nature of the defect emitting at 880 nm is related to its strong coupling to coherent phonon polariton excitations in the sample. Notably, we also observe a second class of defects in our liquid-exfoliated hBN flakes that emit at 530 nm (zero-phonon line; see Supplementary Fig. 11b). This emission is accompanied by two phonon lines at 553 nm, and 566 nm, indicating an energy difference of 97.3 meV and 158.9 meV between the zero-phonon line and the subsequent phonon lines, respectively. This particular defect has been extensively studied in the literature [11-17] and is known to have a long decay time of a few nanoseconds, in contrast to the defects we have studied so far.

To study this second class of defects in our hBN flakes, we utilize an EDPHS structure with a hole in the center that sustains a broadband spectrum as shown in Supplementary Fig. 4c (see green part of Supplementary Fig. 10b). The CL spectrum of the combination of the hBN and EDPHS is also shown in Supplementary Fig. 10b in red, demonstrating efficient excitation of the emitting center at 530 nm. The spectral profile of the EDPHS structure is perfectly suited for the excitation of the coherent phonon lines at 797 nm and 670 nm. In addition, a faint spectral feature associated with the defect centers emitting at 880 nm is observed, shifted to 860 nm due to the high-intensity phonon lines covering the emitter wavelengths.

Hyperspectral images associated with $\lambda_1 = 530\ \mathrm{nm}$, $\lambda_2 = 670\ \mathrm{nm}$, $\lambda_3 = 797\ \mathrm{nm}$, $\lambda_4 = 860\ \mathrm{nm}$ indicate that a single defect emitting at $\lambda_4 = 860\ \mathrm{nm}$ is effectively excited by the combination of the EDPHS and the hBN flake. However, the CL intensity associated with the emission at $\lambda_1 = 530\ \mathrm{nm}$ is more uniformly distributed within the sample, increasing the efficiency of coupling to this emitter at this sample location.

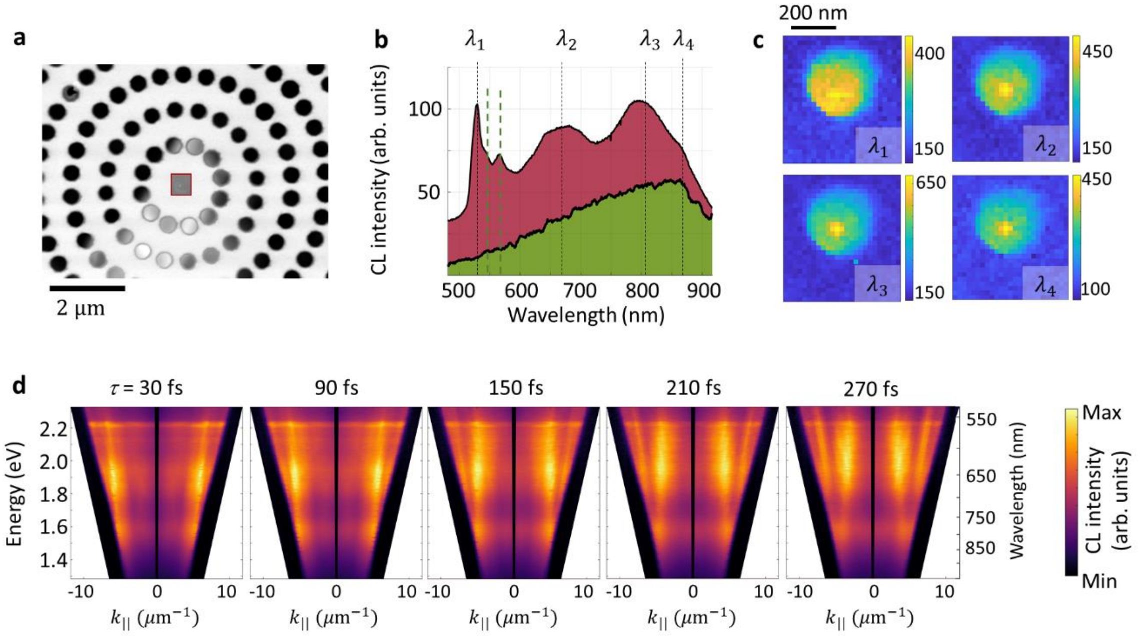

Supplementary Figure 11: Investigation of the dephasing time of the defect emitting at $\lambda = 530\ nm$. (a) The secondary electron dark-field image of the EDPHS and an hBN flake below it, positioned 20 μm away from the EDPHS. The electron beam is focused on the EDPHS. (b) The CL spectra of the EDPHS (green shaded area) and the combination of EDPHS and sample integrated over the shaded box in the panel (red shaded area) (a). (c) Hyperspectral CL images of the combination of the EDPHS and hBN flake at the wavelength shown. (d) Momentum-resolved CL spectral maps at the indicated delays between the EDPHS and the sample radiation.

This emitter is not coupled to coherent phonon lines at $\lambda_2 = 670$ nm and $\lambda_3 = 797$ nm. This is more evident from the momentum-resolved CL spectral maps. The interference fringes observed in the experiments reported in Figure 3 of the main text are absent from the acquired CL energy-momentum maps shown in Supplementary Figure 10d, highlighting the lack of a coherent emission mechanism from these defects.

Supplementary Note 5. Phonons and Phonon-Polaritons in HBN Flakes

Finally, we give an overview of phonon-polaritons excited in our hBN flakes. Within two frequency ranges in the Reststrahlen bands, hBN hosts hyperbolic phonon polaritons. The permittivity of the material along the in-plane and out-of-plane directions has opposite signs in the regions highlighted in Supplementary Fig. 12 a, leading to different type I and II hyperbolic responses for the regions within $\hbar\omega_{\mathrm{TO},\perp} = 0.097\,\mathrm{eV}$ and $\hbar\omega_{\mathrm{LO},\perp} = 0.103\,\mathrm{eV}$ (shaded in green), and $\hbar\omega_{\mathrm{TO},\parallel} = 0.17\,\mathrm{eV}$ and $\hbar\omega_{\mathrm{LO},\parallel} = 0.20\,\mathrm{eV}$ (shaded in red), respectively[14]. The former and the latter are attributed to the lower and upper Reststrahlen bands, respectively. Given the growth conditions of the flakes, the in-plane and out-of-plane directions correspond to the parallel and the normal directions, respectively, relative to the surface of the flakes.

The electron beam can excite bulk LO and TO excitations in the lower Reststrahlen band as it traverses the hBN flake in the direction normal to the flake (see Supplementary Fig. 12b), resulting in two electron energy-loss peaks at the corresponding energies. However, hBN films cannot host any guided waves or surface phonon polaritons in the lower Reststrahlen band. Notably, the energy shift of the first phonon line corresponding to the lower wavelength emitters is in good agreement with $\hbar\omega_{\mathrm{TO},\perp} = 0.097\,\mathrm{eV}$.

We conclude that these classes of emitters efficiently couple to phonons in the lower Reststrahlen band.

In contrast, in the upper Reststrahlen band, hBN films as thin as 10 nm can host hyperbolic phonon polaritons. The dispersion of these polariton waves is captured in the calculated momentum-resolved electron energy-loss spectra (Supplementary Fig. 12c). In addition to phonon polaritons, a distinct signature is observed at $\hbar\omega_{\mathrm{LO},\parallel} = 0.20\,\mathrm{eV}$, corresponding to the excitation of bulk LO phonons. Finally, when considering bulk excitations (boundary effects are neglected), a clear signal is observed in the calculated momentum-resolved electron energy-loss spectra, corresponding to the Cherenkov radiation (Supplementary Fig. 12d).

The lower Reststrahlen band is hardly accessible due to the broadening of the zero-loss peak, when performing low-energy electron energy-loss spectroscopy. However, as shown in the main text, phonon-polariton excitations in the upper Reststrahlen band is clearly accessible with this technique. To better determine the excitation of both phonon-polaritons and bulk LO phonons, low-energy electron energy-loss spectra are now acquired in an aloof excitation, where bulk phonons are not excited, and the results are compared to the case where the electron beam traverses the flake (Supplementary Fig. 13). Even in vacuum and at a distance of 10 nm from the flake, electron beams can excite surface phonon polaritons, leading to a sharp peak at the energy of $E_2 = 0.186$ eV corresponding to surface phonon polariton excitation, where the strongest signal is also observed in simulations (compare Supplementary Fig. 13a with Supplementary Fig. 12c). Bulk LO phonons also appear in the EELS signal when the electron beam traverses the material at a given thickness (Supplementary Fig. 13 b and c). A lower energy excitation is also observed at 0.157 meV, which is below the upper Reststrahlen band and corresponds to Cherenkov radiation.

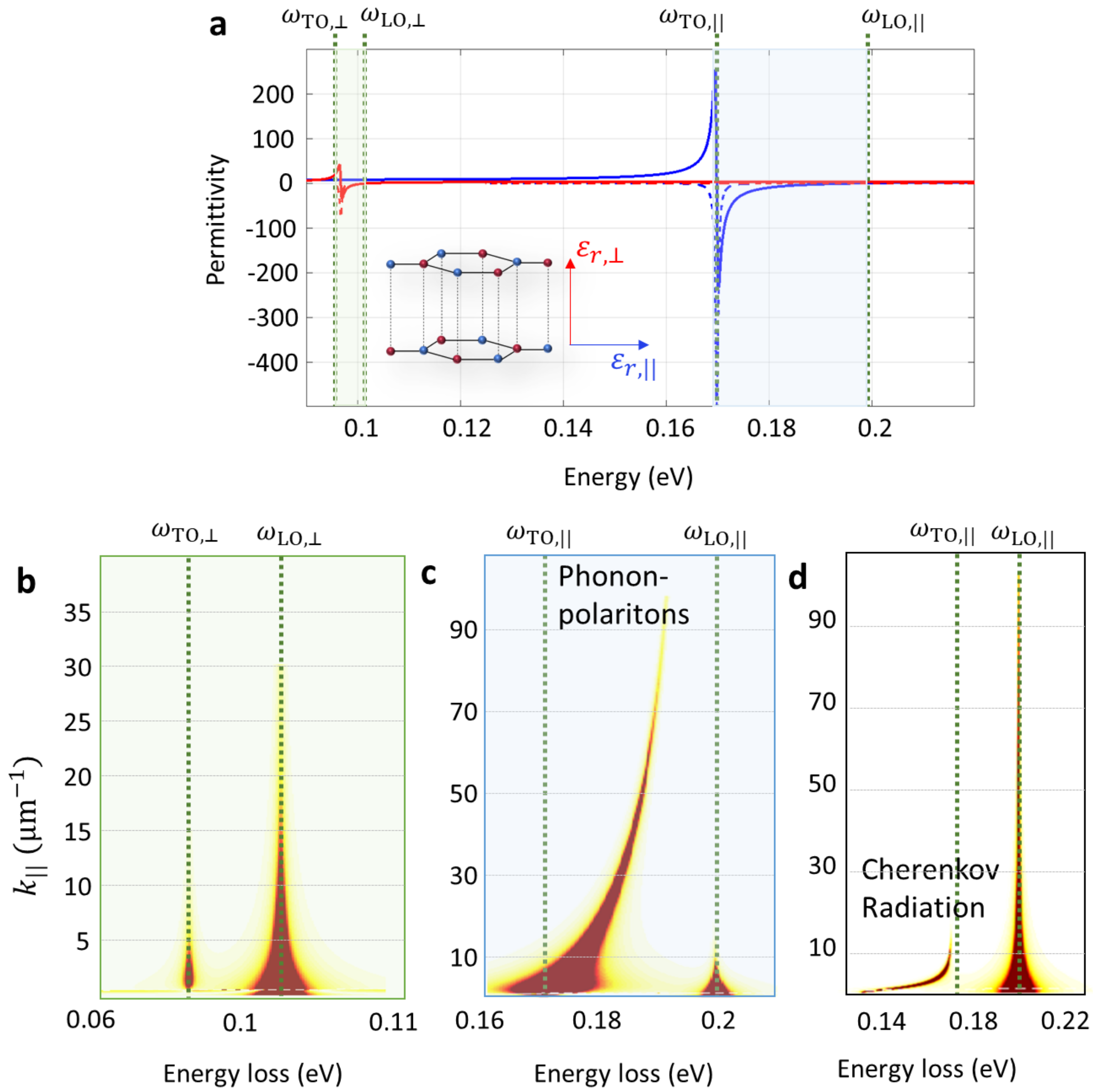

Supplementary Figure 12: Phonon-polaritons in hBN. (a) Permittivity of hBN, demonstrating the anisotropic nature of the materials and its hyperbolic behavior within the two Reststrahlen bands. The real and imaginary parts of the permittivity are indicated by solid and dashed lines, respectively. The atomic structure of the material is shown in the inset. The in-plane and out-of-plane permittivity components are shown as blue and red lines, respectively. (b - d) Calculated momentum-resolved electron energy-loss spectra in the (b) lower and (c) upper Reststrahlen bands and in a bulk hBN medium without considering the boundary effects. The electron has a kinetic energy of 30 keV and traverses an hBN thin film in the direction normal to the surface of the flake. The thickness of the flake is 10 nm.

The energy of the first phonon line, corresponding to the emitters emitting at 880 nm, exhibits an energy shift of about 0.147 eV, with respect to the zero-phonon line. However, this peak has a wide bandwidth, so that the energy shift with respect to the zero-phonon line ranges from 0.11 eV to 0.22 eV. This energy shift is significantly larger than $\hbar\omega_{\mathrm{TO},\perp} = 0.097\,\mathrm{eV}$ and more pronounced than the upper Reststrahlen band. However, due to the clear signals observed in the low-energy electron energy-loss spectra corresponding to the Cherenkov radiation, we would not rule out the influence of the Cherenkov radiation in the observed coherent interactions.

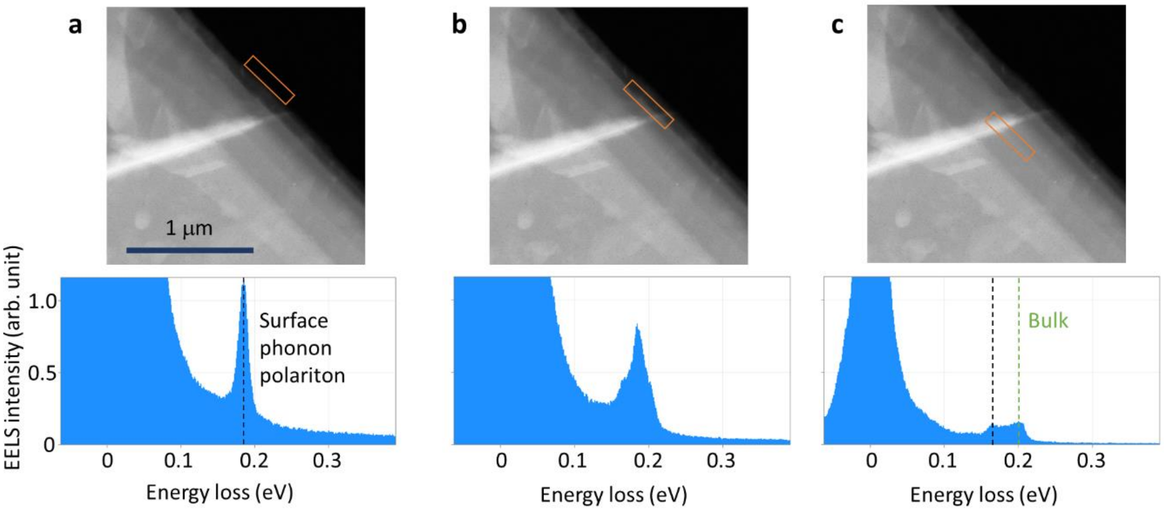

Supplementary Figure 13: Position dependence of the low-energy electron energy-loss spectra. The upper figures are the dark-field TEM images and the lower figures depict the low-energy electron energy-loss spectra at the positions marked by the orange box. (a) Aloof excitation, where only a single peak at $E = 0.186$ eV is observed, corresponding to surface-phonon polaritons scattered from the edge of the flake. (b, c) As electrons traverse the flake, bulk excitations are also excited, resulting in peaks corresponding to LO phonons and also Cerenkov radiation.

Supplementary Note 6. Excitation of multiple defects and energy-exchange rate between emitters

In the case of excitations of multiple emitters in close proximity, the relaxation dynamics are influenced by the coupling between these emitters. The preparation of hBN thin films via liquid exfoliation enables the generation of a large number of emitters within a small region. Notably, the sonication duration plays a significant role in determining the number of emitters produced. However, two types of emitters, emitting at wavelengths of 530 nm and 860 nm, are consistently observed. These findings emphasize the critical influence of free-induction decay and emitter-emitter couplings on the decay time and decoherence dynamics of individual emitters, rather than on the specific type of emitter excited.

Hyperspectral cathodoluminescence imaging serves as a powerful tool for determining the positions and numbers of emitters in our hBN films. Two representative hyperspectral images are shown in Supplementary Fig. 14. Emitters at $\lambda_1 = 860\,nm$ are less abundant in the flake displayed in Supplementary Fig. 14a compared to the flake shown in Supplementary Fig. 14c (see hyperspectral images at $\lambda_1 = 860\,nm$). In contrast, emitters at $\lambda_4 = 530\,nm$ are more numerous in this flake.

Emitters at $\lambda_1 = 860\,nm$ are coupled to coherent phonons, resulting in relaxation dynamics that are significantly influenced when many emitters are positioned in close proximity. This is attributed to direct phonon-mediated energy transfer between emitters, as observed for the flake shown in Supplementary Figs. 14c and 14d. Conversely, for the flake shown in Supplementary Fig. 14a, the measured relaxation and decoherence time scales are 510 fs and 180 fs, respectively. These values are comparable to the dynamics of the emitter discussed in the main text.

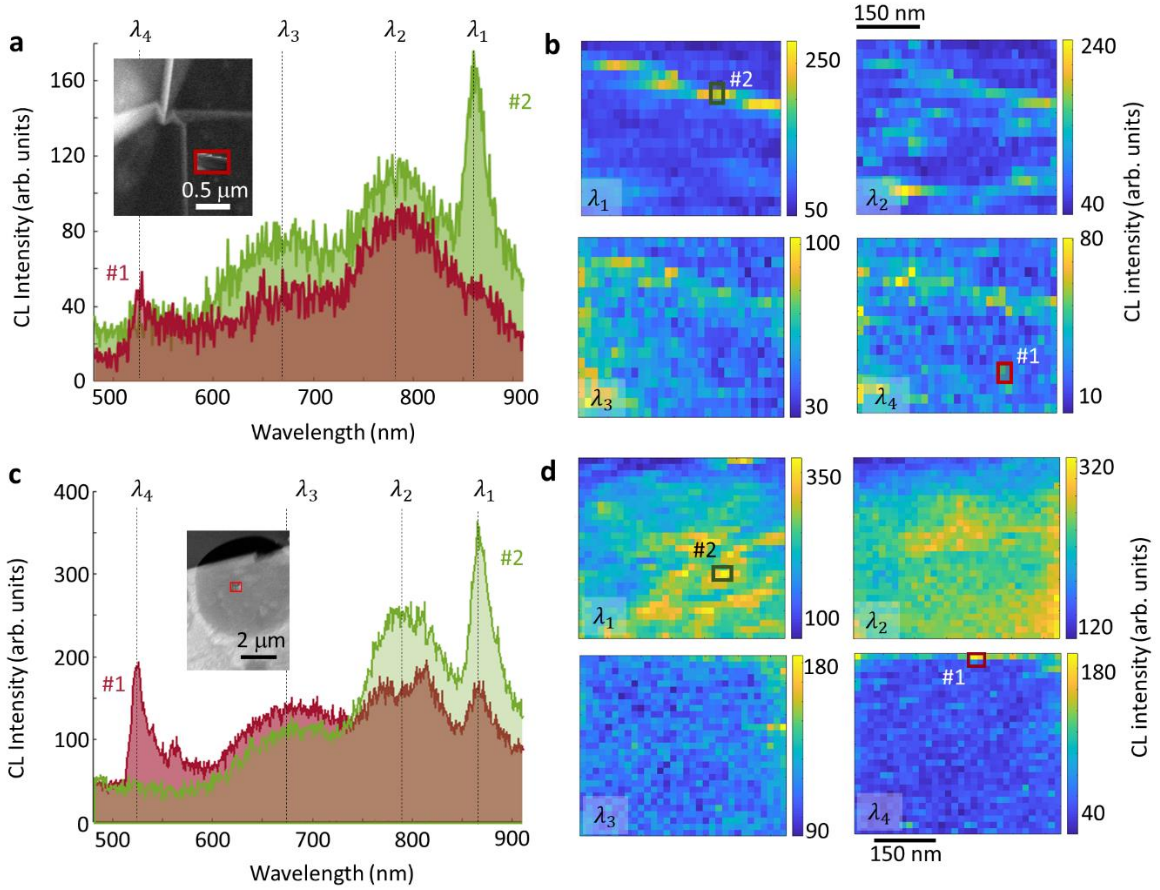

Supplementary Figure 14: Statistics of emitters in hBN thin films. (a, c) CL spectra and (b, d) hyperspectral images, at depicted wavelengths of λ_1 to λ_4 , of two hBN films prepared using liquid exfoliation, positioned on the holey carbon substrate. The explored regions are suspended in vacuum. The secondary-electron SEM images of the hBN flakes are provided in the insets of panels (a) and (c).

Using the EDPHS structure shown in Supplementary Figs. 4 and 5, we examined the relaxation and decoherence dynamics of various hBN flakes. The results presented in Supplementary Fig. 15 specifically correspond to the flakes shown in Supplementary Figs. 14a and 14b, focusing on the electron impact position marked as #2 for both flakes.

For the first flake, the population relaxation dynamics were determined by fitting an exponential function to the peak intensities, yielding a relaxation time of approximately 510 fs. In contrast, for the second flake shown in Supplementary Figs. 14c and 14d, where a higher density of emitters is excited, the coupling between emitters was characterized by fluctuations in the cathodoluminescence (CL) emission intensity as a function of the delay between the EDPHS-generated pulses and the arrival of the electron beam at the sample.

The overall relaxation time in the second flake is influenced by free-induction decay between emitters, which also contributes to faster decoherence dynamics and population relaxation. The accelerated decoherence dynamics are evident in the angle-resolved map acquired at a wavelength of $\lambda_1 = 850\, nm \pm 25\, nm$ (Supplementary Fig. 15c), where the visibility of the interference fringes diminishes within 60 fs.

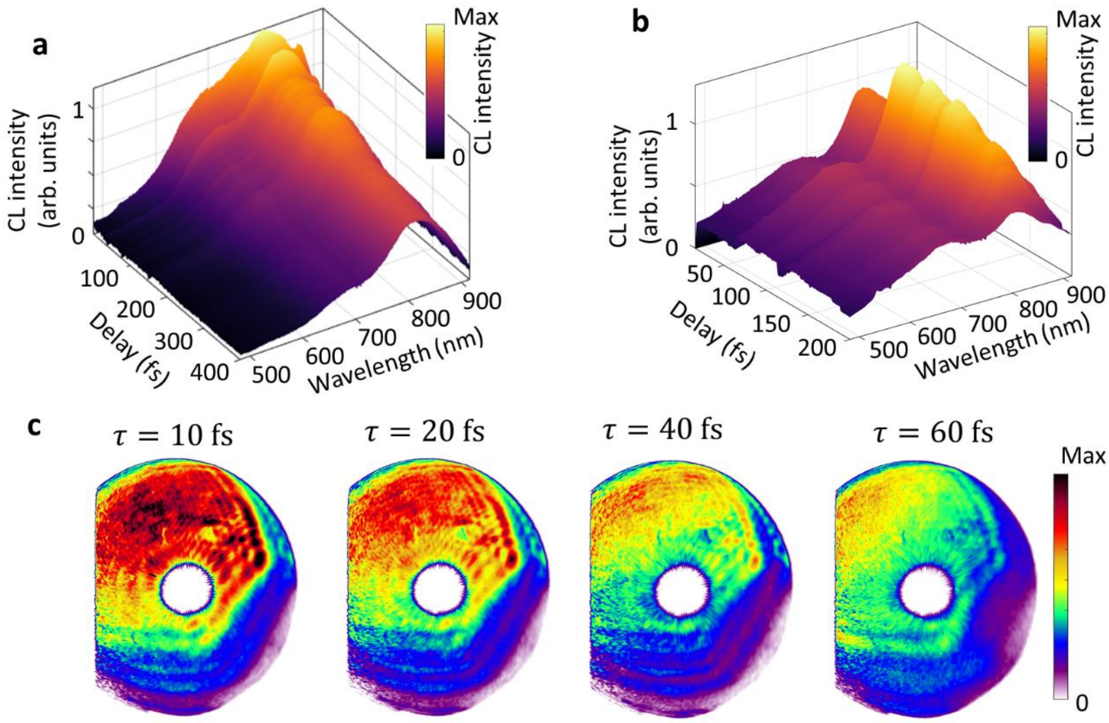

Supplementary Figure 15: Relaxation dynamics of the emitters at the positions marked by #2 in Supplementary Fig. 14. (a, b) Population decay demonstrated by acquiring the CL spectra versus the delay between the EDPHS radiation and electrons arriving at the sample. (a) and (b) correspond to the flakes shown in Supplementary Fig. 14 a and c insets, respectively. (c) Angle-resolved CL maps at depicted delays between electrons and EDPHS radiation, for the flake shown in Supplementary Figure 14 (c).

References

1 in *Decoherence and the Quantum-To-Classical Transition* 293-328 (Springer Berlin Heidelberg, 2007).

2 Yuge, T., Yamamoto, N., Sannomiya, T. & Akiba, K. Superbunching in cathodoluminescence: A master equation approach. *Physical Review B* **107**, 165303 (2023). https://doi.org:10.1103/PhysRevB.107.165303

3 Loudon, R. *The Quantum Theory of Light*. 52 (Oxford University Press, 2010).

4 Groll, D., Hahn, T., Machnikowski, P., Wigger, D. & Kuhn, T. Controlling photoluminescence spectra of hBN color centers by selective phonon-assisted excitation: a theoretical proposal. *Materials for Quantum Technology* **1**, 015004 (2021). https://doi.org:10.1088/2633-4356/abcbeb

5 Morse, P. M. Diatomic Molecules According to the Wave Mechanics. II. Vibrational Levels. *Physical Review* **34**, 57-64 (1929). https://doi.org:10.1103/PhysRev.34.57

6 Fues, E. Das Eigenschwingungsspektrum zweiatomiger Moleküle in der Undulationsmechanik. *Annalen der Physik* **385**, 367-396 (1926). https://doi.org:https://doi.org/10.1002/andp.19263851204

7 Christopher, J. *et al.* Electron-driven photon sources for correlative electron-photon spectroscopy with electron microscopes. **9**, 4381-4406 (2020). https://doi.org:doi:10.1515/nanoph-2020-0263

8 Talebi, N. *et al.* Merging transformation optics with electron-driven photon sources. *Nature Communications* **10**, 599 (2019). https://doi.org:10.1038/s41467-019-08488-4
9 van Nielen, N. *et al.* Electrons Generate Self-Complementary Broadband Vortex Light Beams Using Chiral Photon Sieves. *Nano Letters* **20**, 5975-5981 (2020). https://doi.org:10.1021/acs.nanolett.0c01964
10 Taleb, M., Hentschel, M., Rossnagel, K., Giessen, H. & Talebi, N. Phase-locked photon–electron interaction without a laser. *Nature Physics* **19**, 869-876 (2023). https://doi.org:10.1038/s41567-023-01954-3
11 Tran, T. T., Bray, K., Ford, M. J., Toth, M. & Aharonovich, I. Quantum emission from hexagonal boron nitride monolayers. *Nature Nanotechnology* **11**, 37-41 (2016). https://doi.org:10.1038/nnano.2015.242
12 Tran, T. T. *et al.* Robust Multicolor Single Photon Emission from Point Defects in Hexagonal Boron Nitride. *ACS Nano* **10**, 7331-7338 (2016). https://doi.org:10.1021/acsnano.6b03602
13 Tran, T. T. *et al.* Deterministic Coupling of Quantum Emitters in 2D Materials to Plasmonic Nanocavity Arrays. *Nano Letters* **17**, 2634-2639 (2017). https://doi.org:10.1021/acs.nanolett.7b00444
14 Boll, M. K., Radko, I. P., Huck, A. & Andersen, U. L. Photophysics of quantum emitters in hexagonal boron-nitride nano-flakes. *Opt. Express* **28**, 7475-7487 (2020). https://doi.org:10.1364/OE.386629
15 Grosso, G. *et al.* Low-Temperature Electron–Phonon Interaction of Quantum Emitters in Hexagonal Boron Nitride. *ACS Photonics* **7**, 1410-1417 (2020). https://doi.org:10.1021/acsphotonics.9b01789
16 Grosso, G. *et al.* Tunable and high-purity room temperature single-photon emission from atomic defects in hexagonal boron nitride. *Nature Communications* **8**, 705 (2017). https://doi.org:10.1038/s41467-017-00810-2
17 Shevitski, B. *et al.* Blue-light-emitting color centers in high-quality hexagonal boron nitride. *Physical Review B* **100**, 155419 (2019). https://doi.org:10.1103/PhysRevB.100.155419
